# Design and Fabrication Challenges of a Highly Sensitive Thermoelectric-Based Hydrogen Gas Sensor

**DOI:** 10.3390/mi10100650

**Published:** 2019-09-27

**Authors:** Anmona Shabnam Pranti, Daniel Loof, Sebastian Kunz, Volkmar Zielasek, Marcus Bäumer, Walter Lang

**Affiliations:** 1 IMSAS—Institute for Microsensors, -actuators and -systems, University of Bremen, 28359 Bremen, Germany; wlang@imsas.uni-bremen.de; 2 IAPC—Institute of Applied and Physical Chemistry, University of Bremen, 28359 Bremen, Germany; daniel.loof@uni-bremen.de (D.L.); sebkunz@uni-bremen.de (S.K.); zielasek@uni-bremen.de (V.Z.); mbaeumer@uni-bremen.de (M.B.)

**Keywords:** thermopile, thermoelectric, hydrogen, gas sensor, silicon nitride, membrane, high sensitivity, temperature field

## Abstract

This paper presents a highly sensitive thermoelectric sensor for catalytic combustible gas detection. The sensor contains two low-stress (+176 MPa) membranes of a combination of stoichiometric and silicon-rich silicon nitride that makes them chemically and thermally stable. The complete fabrication process with details, especially the challenges and their solutions, is discussed elaborately. In addition, a comprehensive evaluation of design criteria and a comparative analysis of different sensor designs are performed with respect to the homogeneity of the temperature field on the membrane, power consumption, and thermal sensitivity. Evaluating the respective tradeoffs, the best design is selected. The selected sensor has a linear thermal characteristic with a sensitivity of 6.54 mV/K. Additionally, the temperature profile on the membrane is quite homogeneous (20% root mean standard deviation), which is important for the stability of the catalytic layer. Most importantly, the sensor with a ligand (p-Phenylenediamine (PDA))-linked platinum nanoparticles catalyst shows exceptionally high response to hydrogen gas, i.e., 752 mV at 2% concentration.

## 1. Introduction

The new technological advancement of micro fabrication has opened a platform for research and development to improve the performance and the characteristics of micro sensors for different applications such as combustible catalytic gas sensing [1,2,3]. A micro-machined thermal sensor with a stable membrane has become the focal point because of its small size and low power consumption [1,2,4,5,6]. Along with that, the sensors are expected to have perfect temperature homogeneity for the long-term stability of the catalyst, high thermal and mechanical strength to intensify the robustness in harsh environments, and compatibility with a standard microfabrication process [1]. Thus, the investigation of the design criteria and dealing with the power loss and fabrication challenges are very important to achieve a robust and highly sensitive thermoelectric catalytic gas sensor. As examples of the design challenges, we note that: (1) a large membrane reduces the conduction heat loss but increases the convection heat loss, (2) a higher number of thermocouples increases not only the sensitivity, but also the conduction heat loss, (3) although a smaller width of a thermocouple reduces power consumption, it may increase the ohmic voltage drop in the thermopile during the signal extraction. Literature on the combustible gas sensor has mainly focused on factors that affect the power consumption [7]. Although Tajima et al. tried to reduce the power consumption of their thermoelectric hydrogen gas sensor by using a suspended micro heater, the sensor consumes 34 mW at 100 °C operating temperature and, moreover, suffers from poor stability and homogeneity of the temperature field [1,4]. Behera et al. prepared a micro gas sensor with a thin silicon diaphragm and a bridge structure of a Ni micro heater that consumes 36 mW at 100 °C temperature [8]. In comparison, the resistance temperature detector (RTD) catalytic gas sensor presented by Trochimczyk et al. consumes only 2 mW power for a pulsating supply [6]. Although an RTD sensor has low power consumption, it shows instability in the output at high operating temperatures and, additionally, a calibration is needed before each operation [6,9]. The power consumption of the sensor depends not only on the size, material, and on the type of the sensor, but also on the heat transfer, which varies for different designs. A comparative analysis of different designs of the thermoelectric sensor based on the heat transfer provides an opportunity to select the optimized design.

Only few researchers have highlighted another important design criterion for the catalytic gas sensors, namely, the homogeneity of the temperature field on the membrane, and have applied ideas such as placing a thin silicon island underneath the membrane [7] that increases the fabrication complexity and power consumption. Conversely, a homogeneous temperature field can also be achieved by optimizing the heater design while maintaining the lowest possible power consumption.

There have been long-standing investigations to improve the sensitivity of thermoelectric sensors by varying the design criteria. Researchers tried different combinations of the substrate materials, thermoelectric materials, and structural designs to affect the thermopile output voltage and to increase the sensitivity of the sensor [5,10,11]. The figure of merit (ZT) of the thermoelectric generator depends not only on the material properties, but also on the operating temperature [12,13]. The sensor temperature may vary for different designs due to different heat transfer phenomena, although the input power is the same. The investigation of this effect on the thermopile sensitivity is interesting in the optimization of the sensor design.

Another key aspect of a highly sensitive and stable micro gas sensor is the fabrication process. Several studies were conducted on the diversity and compatibility of the micro-fabrication processes for producing a desired sensor design. Fardindoost et al. presented an RTD hydrogen gas sensor with Pd-doped WO_3_ films on Al_2_O_3_ substrates [14] that needs to be calibrated before use, and the base resistance of the thermometer may change with the operating temperature [15]. Unlike the RTD sensor, the thermoelectric sensor directly converts the temperature change into the voltage during the combustion reaction, and no calibration is needed [9,16,17]. In fact, the thermoelectric material plays a major role for the sensitivity of the sensor. Researchers have introduced several thermoelectric materials with high Seebeck coefficients such as Bi_2_Te_3_ (−188.5 µV/K, n-type and +160 µV/K, p-type)_,_ Bi_2_Te_3_ based alloy (−179.3 µV/K, n-type and +254.4 µV/K, p-type), n-type SiGe (−77 µV/K to −198 µV/K), and polysilicon (−57 µV/K, n-type and +103 µV/K, p-type) [12,13,16,18,19]. Tajima et al., Matsumiya et al., Nagai et al., Shin et al., and Goto et al. presented thermoelectric gas sensors with SiGe, Li-doped NiO, alkali-doped NiO, and p-type BiSbTe thermoelectric elements, respectively [16,20,21,22,23]. The selection of the material should be based not only on a high Seebeck coefficient, but also on the compatibility with the following fabrication process and the operating temperature of the sensor [12]. In this work, we used a combination of polysilicon and tungsten titanium that remained completely stable during the successive high temperature fabrication processes and showed quite a high Seebeck coefficient, i.e., +273 µV/K. A chemically and thermally highly stable membrane is fabricated by a composition of stoichiometric and low-stress SiN at very high temperature. Not only the material, but also the small steps in between the consecutive fabrication processes, are dominant factors for producing a highly sensitive thermoelectric gas sensor. For an example, if a subsequent layer is deposited onto the residual photoresist, the contact resistance between the layers may increase and ultimately limit the quality of the sensor. While the existing literature on the fabrication of thermoelectric gas sensors is extensive and focuses particularly on the main fabrication processes, small details are missing. Almost no literature has pointed out the small problems and the failures confronted during the development of a fabrication process and the solutions to these problems before coming out with a successful highly sensitive sensor. These small details of the fabrication processes are of particular interest to the community of researchers to replicate the work and/or to use this knowledge for the improvement of their own research.

The motivation of the present work is to design a new sensor with an excellent temperature uniformity on the membrane by optimizing the heater structure along with the lowest possible power consumption and the highest thermopile output. It is difficult to predict the heat loss for a given micro sensor design [21]. Therefore, sensors of different designs were fabricated and the respective main challenges are discussed in the following pages. The thermal characteristics of the different designs are analyzed qualitatively as well as quantitatively, and the best design is selected based on temperature homogeneity, power consumption, and the sensitivity. Additionally, this paper presents the complete fabrication process of this highly sensitive thermoelectric sensor in detail, along with the problems that were encountered during processing and their solutions. These should be particularly useful to the growing research community in the field of MEMS-based gas sensor fabrication.

## 2. Materials and Methods 

### 2.1. The Design of the Sensor

**Figure 1:**
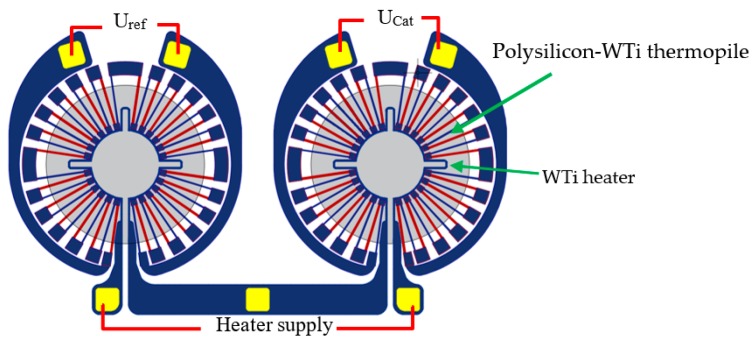
The schematic of the sensor design.

The sensor contains two circular membranes of 900 µm diameter (a catalytic membrane and a reference membrane) and two thermopiles (24 thermocouples connected in series in each) of a combination of WTi-polysilicon encircling the membrane, as shown in the Figure 1. The width of the WTi and polysilicon thermoelectric elements is 12 µm and 8 µm respectively. The three contact points of the heater facilitates the use of an individual power supply and temperature controlling. The hot junctions of the thermocouple are placed very close to the heater, i.e., 5 µm apart. This is because the closer the thermoelectric elements and the heater are to one another, the is the heat loss and the better the sensitivity [16,24]. Conversely, the hot and the cold junctions of the thermopiles are placed as far apart from each other as possible to improve the thermoelectric performance [16]. For this reason, a meander-shaped heater is avoided. According to the main idea of the sensor, the hydrogen combustion reaction occurs only on the membrane on which the catalyst is. The temperature difference between the hot and the cold junctions of the catalytic thermopile increases due to the heat of the reaction that increases the thermoelectric voltage (Ucat). Conversely, the thermopile voltage of the reference membrane (Uref) remains constant unless an environmental change (such as a change of the gas flow rate) occurs which alters the heat balance between the hot and cold junctions of the membranes. The output of the sensor is the difference between the two thermopile voltages.

### 2.2. Fabrication Challenges of the Sensor and Solutions

**Figure 2:**
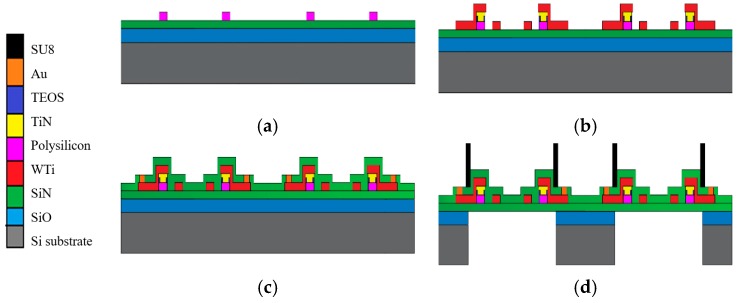
Schematic diagram of the fabrication steps of the sensor, after (**a**) the deposition of silicon oxide, first phase of SiN and structuring of polysilicon layers; (**b**) the structuring of TEOS, TiN, WTi layers; (**c**) deposition of the second phase of SiN layer; (**d**) deposition and structuring of SU8 layer and backside (Si) structuring (membrane opening) [31] (Reproduced with permission from Pranti, A.; Loof, D.; Kunz, S.; Zielasek, V.; Bäumer, M.; Lang, W., Ligand-Linked Nanoparticles-Based Hydrogen Gas Sensor with Excellent Homogeneous Temperature Field and a Comparative Stability Evaluation of Different Ligand-Linked Catalysts; Published by MDPI, 2019).

**Figure 3:**
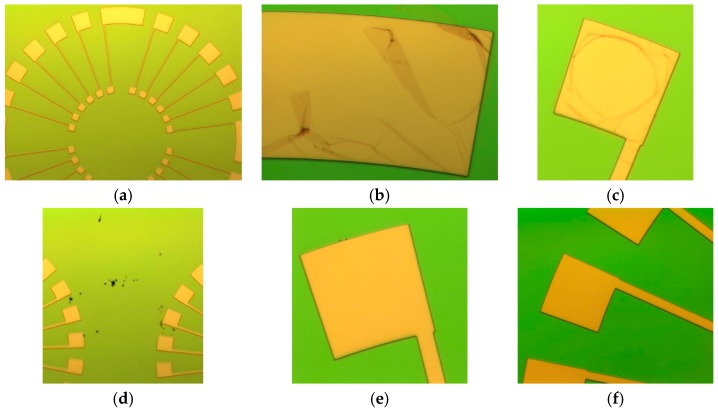
(**a**) Very small structure (4 µm) that resolved after RIE of polysilicon; (**b**) and (**c**) residue of photo resists after RIE of polysilicon and removing of photoresist by the standard process with AZ 100 solution; (**d**) burnt photoresist after RIE of polysilicon; (**e**) and (**f**) photoresist residue completely cleaned after 7 min of oxygen plasma.

Figure 2a–d represents the schematic of the sensor fabrication steps chronologically. The 2 mm × 3 mm sensor was fabricated on a 380 µm-thick, 100 mm diameter, double-side polished silicon wafer. At the beginning, the wafer was cleaned in Caro’s acid, a solution of 30% hydrogen peroxide (H_2_O_2_) and 96% sulfuric acid (H_2_SO_4_) of equal volume, for 15 min to remove any organic substances [25]. After that, a 500 nm-thick thermal silicon oxide (SiO_2_) was grown in a wet atmosphere at 1000 °C. The thermal oxide layer serves two purposes: (1) it works as an etch stop layer during the deep reactive ion etching of the Si wafer to form the membranes, and (2) it acts as a thermal insulation layer between the silicon substrate and the membrane material. The 600 nm-thick silicon nitride (SiN) membrane was fabricated in two phases. The first phase of the layer was deposited on top of the thermal oxide layer by an LPCVD process at 800 °C with dichlorosilane (H_2_SiCl_2_) and ammonia (NH_3_) gases. This layer is a composition of stoichiometric and low-stress SiN; the low-stress SiN layer is sandwiched in between two stoichiometric layers. A stoichiometric SiN layer was deposited for 10 min, followed by a low-stress, silicon-rich layer for 125 min and a stoichiometric layer for 2 min. The average thickness and tensile stress of the layer are 304.7 ± 3.8 nm and 176.5 ± 13.2 MPa, respectively. This LPCVD low-stress SiN creates a high quality, chemically- and mechanically-stable, freestanding membrane. Afterwards, the first thermopile material, a 328.7 ± 11.8 nm-thick p-doped polycrystalline silicon layer, was formed by a LPCVD process at 670 °C by supplying silane (SiH_4_) for 110 min. The average sheet resistivity (right after the deposition of the layer) was 354 ± 16 Ω (commonly used unit Ω/sq). As the polysilicon is oxidized quite easily, silanol (SiOH) is formed on the surface with the help of the environmental water molecules, and the photoresist cannot adhere perfectly [26,27]. Therefore, an adhesion promoter (HMDS) was used and deposited by evaporation in an oven at 140 °C before polysilicon structuring. A 1.8 µm-thick photoresist, AZ 1518 from MicroChemicals GmbH, Ulm, Germany, was used throughout the work unless stated otherwise. The photoresist should be coated right after the deposition of the HMDS, because it was seen during our process that waiting too long between the HMDS deposition and photoresist coating (45 min.) caused deterioration of the adhesion to occur. The polysilicon layer was structured as the first thermopile element by reactive ion etching (RIE) for 1 min 15 s with the coil and a RF power of 60 W and 5 W respectively. That resulted in a high spatial resolution [28], less than 0.5 µm under etching; Figure 3a shows that 4 µm wide structures were also perfectly resolved. SF_6_ and N_2_ gases were used at 30 sccm and 60 sccm flow rates for the chemical and the physical etching, respectively, at a chamber pressure of 5 mTorr. The polysilicon layer from the backside of the wafer was removed in the same way. After etching, the 1.8 µm photoresist was removed by a photoresist remover (AZ 100 of MicroChemicals GmbH, Ulm, Germany) in a standard process for 15 min at 50 °C, although some photoresist residue remained on some of the structures; see Figure 3b,c. Some burnt photoresists were also found, as shown in Figure 3d (red circle). To solve these problems, acetone and isopropanol, VLSI grade, were applied to the wafer on a spin coater that reduced the photoresist residue, albeit incompletely. Therefore, oxygen plasma was applied on the wafer afterwards at 500 W, as a solution by which to remove carbon or photoresist residue [29,30]. Several consecutive oxygen plasma processes of 1 min duration were used to obtain the optimum result, and the wafers were investigated optically after the etching process. Figure 3e,f indicate that the wafers were completely free of photoresist residue after 7 min of oxygen plasma treatment.

Annealing the thermoelectric film at a high temperature improves the quality by reducing defects and pinholes in the film [12,19,32,33]. Annealing reduces the electrical resistance of the layer, thus reducing the signal-to-noise ratio (SNR) during the acquisition of the thermoelectric voltage [33]. Therefore, the structured polysilicon layer was annealed at 930 °C in a N_2_ gas environment for 120 min. The resistivity of the polysilicon layer after annealing was around 7.4 ± 0.3 mΩ·cm. 

**Figure 4:**
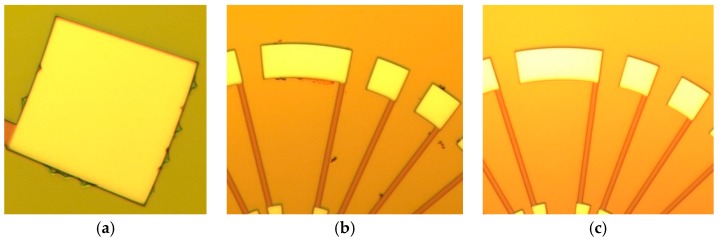
After silicon oxide (TEOS) etching, (**a**) and (**b**) the residual photo resist after using of 1 min O_2_ plasma and standard AZ 100 photoresist remover; (**c**) cleaned surface after using 1 min O_2_ plasma, AZ 100 remover and further 3 min O_2_ plasma.

A very thin silicon oxide layer, i.e., 72.8 ± 1.9 nm, was used on the polysilicon as the etch stop layer for structuring of the next titanium nitride (TiN) layer. The benefit of using silicon oxide as an etch stop was investigated beforehand by applying the TiN etching process in Cl_2_ and Ar gases to a polysilicon and a silicon oxide layer. There, it was found that 194 nm polysilicon and only 13.9 nm oxide layer were etched over the same etching time (1 min 30 s). The etch stop oxide layer was deposited in a LPCVD process at 800 °C by a chemical reaction between TEOS (Si(OC_2_H_5_)_4_) and oxygen (O_2_) [34,35] because thermal oxidation at a very high temperature could affect the polysilicon layer underneath. An opening of the polysilicon layer was made for the contact to the next TiN layer by RIE etching the oxide layer for 1 min 15 s in CF_4_ and CHF_3_ gases at 15 sccm and 10 sccm flow rate respectively. The etching was performed at a chamber pressure of 5 mTorr, a coil power 800 W, and a RF power 50 W. Additionally, an oxygen plasma for 1 min was done at 500 W, right after the etching and the photoresist were removed by the aforementioned standard process that left small amounts of residue at the edge of the structures, as shown in the Figure 4a,b. A further oxygen plasma for 3 min cleaned the residual resist perfectly, as shown in the Figure 4c.

**Figure 5:**
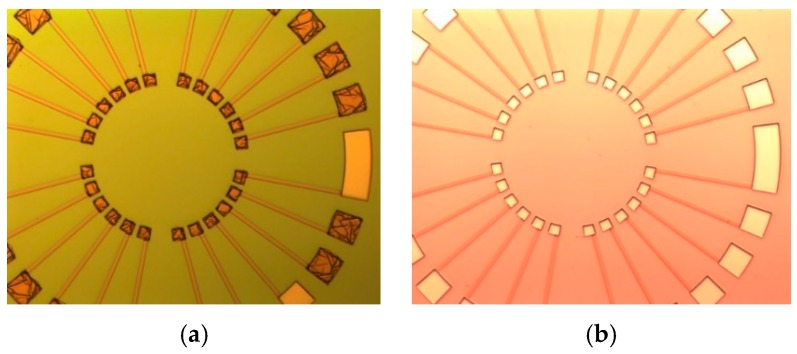
After RIE of TiN and removing of photoresist (**a**) burnt photoresist stuck on the surface when 1.8-µm photoresist was used and removed in AZ 100 and DMP solution; (**b**) perfectly cleaned surface when 6.5-µm photoresist was used and cleaned in AZ 100 with 30 s prior oxygen plasma.

The successive high temperature process may initiate the formation of silica on the polysilicon that reduces the effective thickness of the thermoelectric conduction area, and hence, the performance of the thermopile [36]. Therefore, a 60 nm-thick titanium nitride (TiN) layer was reactively sputtered by 400 W RF power in N_2_-Ar atmosphere to prevent the silica formation by diffusion. The chamber was cleaned by Ar flash at 90.2 sccm and evacuated at 8 × 10^−7^ mbar along with a pre-sputter etching beforehand. The layer was structured by RIE in Cl_2_ and Ar flow at 50 sccm and 10 sccm respectively for 1min 30 s at 5 mTorr chamber pressure with 800 W coil power and 15 W·RF power. Surprisingly, the photoresist seemed burnt, broken and distorted, as shown in Figure 5a, after applying the AZ 100 standard photoresist removing process (as stated above). To solve this problem, DMP (dipropyleneglycol-monomethyl ether) solvent was used, because it can partially dissolve burnt photoresists [37]. The wafer was kept in a 500 mL, heated (65 °C) DMP solution for 15 min, but almost no improvement was seen. An alternative approach, i.e., applying Caros’s acid, removed not only the burnt photoresist but also the underneath TiN layer, as it attacks some base metals (Al, Ti, Ni) in addition to organic materials [38,39].

An effective process was developed to solve this problem. A 6.5-µm-thick photoresist, AZ 9260 of MicroChemicals GmbH, Ulm, Germany, was used as a mask instead of a 1.8 µm photoresist. The idea was to remove the thick photoresist like a lift-off process using the AZ 100 photoresist remover. This works because the Cl_2_ gas only attacks the top surface of the photoresist during the TiN etching process; the side walls of the photo resist remain unchanged. Therefore, the photoresist-removing solution can enter through the sidewalls and remove the resist perfectly by a lift-off process. The wafer was heated for 2 min both before and after the coating by 6.5-µm photoresist at 100 °C and 110 °C, respectively. Additionally, an oxygen plasma for 30 s at 500 W was applied right after the etching, and the photoresist was removed immediately by the standard process. As result, the photoresist was removed perfectly, as shown in the Figure 5b.

**Figure 6:**
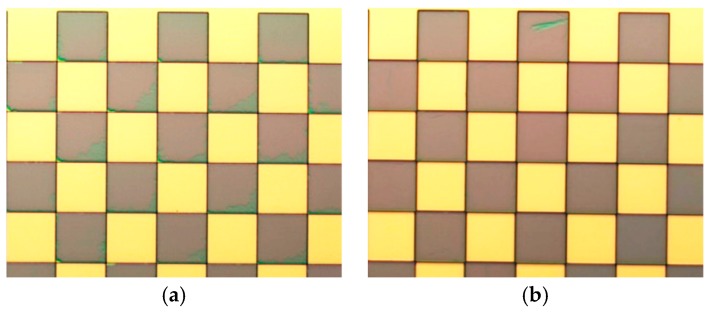
The condition of the test wafers (gray part should be etched) (**a**) after the wet etching of WTi with ‘TiW etch 100’ solution, very thin green layers are left on the gray parts; (**b**) after the wet etching and 30 s O_2_ plasma, the condition is better but still some green layers are left inside the black ring.

Then, a 200 nm-thick tungsten-titanium (WTi) layer was deposited by DC sputtering from a WTi target (90% W and 10% Ti) for 5 min 9 s at 6.9 × 10^−4^ mTorr chamber pressure and 1.5 kW power with a pre-sputter etching. The layer was wet-chemically structured as the second thermopile element by the TiW etch 100 solution of NB Technologies GmbH, Bremen, Germany. The main problem was that after the etching, a very thin layer of WTi stayed on the surface, as shown inside the red ring of Figure 6a; this can create a short circuit among the structures. One possible solution may be using oxygen plasma right after etching. This helps, but it cannot remove the thin layer perfectly, as shown inside the red (cleaned) and black ring (not cleaned) of the Figure 6b.

**Figure 7:**
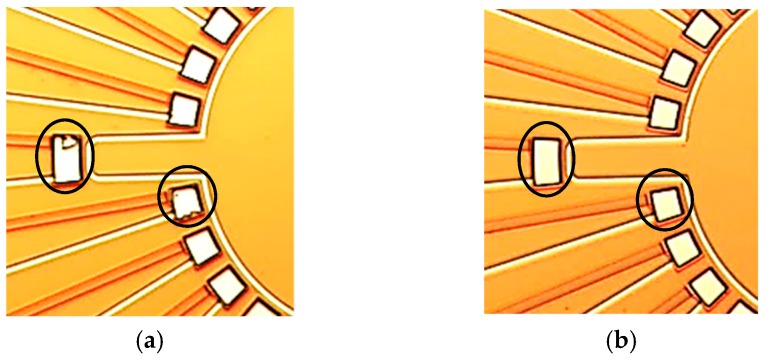
The condition of the structures (**a**) after the standard wet chemical etching by ‘TiW etch 100’ solution, residue left; (**b**) after the wet chemical etching in ‘TiW etch 100’ solution and a deep etching in the photoresist developer AZ 400K, perfectly cleaned.

To solve this problem, wet etching was performed in the ‘TiW etch 100’ solution for 3 min 20 s after exposing the wafer to an oxygen plasma of 30 s at 500 W. Immediately after the etching, a dip etch for 20–30 s was done in the photoresist developer AZ 400K of MicroChemicals GmbH, Ulm, Germany. The entire process was done in a room with dim yellow light to avoid exposing of the photoresist. Figure 7a,b show the condition of the structures when the standard wet chemical etching with ‘TiW etch 100’ and the aforemetioned process were done, respectively. No residue of WTi is seen in the Figure 7b, as in Figure 7a.

After structuring the TiN layer, the underneath silicon oxide (TEOS) layer was removed to avoid stress discrepancy in the membrane. The removal was done wet-chemically using an ‘Oxide etch 7:1 modified’ solution of Honeywell, Germany. In this stage of the fabrication, the top membrane layer (300 nm silicon nitride) was deposited by a LPCVD process. The layer acts as the insulation for the functional layers (heater and thermopiles) and prevents their characteristics from changing during the combustion reaction [40]. To create the layer, a stoichiometric silicon nitride was deposited for 10 min by ramping the temperature from 700 °C to 800 °C, followed by a low-stress silicon nitride for 125 min at 800 °C; the process was concluded with the deposition of a stoichiometric silicon nitride layer for 2 min at 800 °C. After that, 150 µm × 150 µm × 300 nm Au pads were created for the wire bonding by DC sputtering at 100 W and structured wet-chemically by ‘Au etch 200’ solution of nb technologies, Bremen, on the opening of the heater and the thermopiles that were created by RIE of the top insulation (SiN) layer. 

Last but not least, a process was developed to create a 40-µm high SU8 ring with a high spatial resolution on the membrane by optimizing the speed of the spin coater, exposing time, and intensity, development time, pre-, post-, and hard-baking time, and temperature. SU8 3025 of MicroChem, Westborough, MA, USA was spin coated at 1700 rpm for 50 s and prebaked in two steps, at 65 °C for 5 min and at 95 °C for 15 min. The wafer was then exposed for 15 min at 220 mJ/cm^2^ energy intensity in hard contact, followed by two-step post baking—at 65 °C for 1 min and at 95 °C for 5 min—and was developed in a XP-SU8 developer of MicroChem, Westborough, MA, USA for 14 min. Afterwards, the wafer was not rinsed in water but in isopropanol. Additionally, a hard bake was done after development at 170 °C for 5 min, as the structures should remain on the membrane surface.

Finally, the membranes were created by the RIE from the backside with a 10-µm-thick photoresist mask. Both the 600 nm SiN and the 500 nm SiO_2_ were removed by the CF_4_ flow at 60 sccm with 1800 W coil power and 20 W RF power, whereas the deep etching of the Si wafer was done with SF_6_ and C_4_F_8_ gases at 350 sccm and 130 sccm, respectively, at 1800 W coil power. The RF power was continuously switched between 25 W and 60 W during the deep reactive ion etching of the Si wafer (etch rate 5.85 µm/min) with a chamber pressure of 4.5 Pa. The deep reactive ion etching was stopped on the etch stop layer, SiO_2_, beneath the membrane that was removed afterwards wet-chemically by oxide etch modified 7:1 of Honeywell, Germany because the incongruity of the internal stress due the presence of silicon oxide layer can hamper the stability of the membrane [4,16]. At the end, the chips were separated by sawing with a protective layer on the membrane. The process described by Buchner et al. [41] inspired the fabrication process.

### 2.3. Measurement Processes

**Figure 8:**
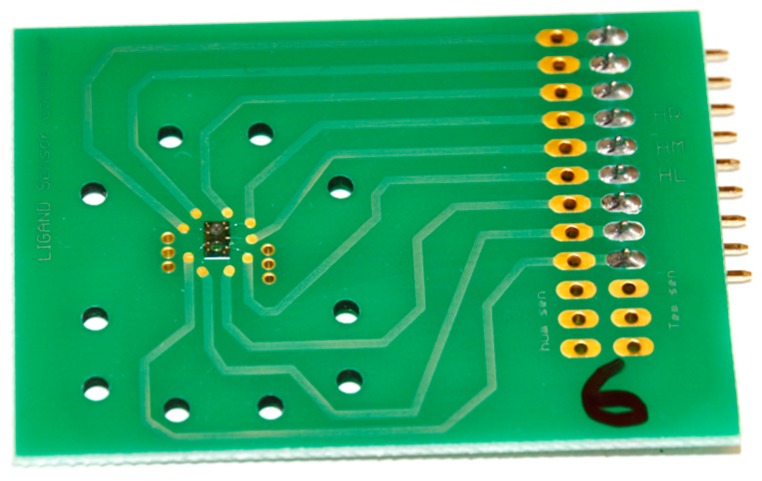
Wire bonded sensor on the PCB.

The sensor was pasted onto a PCB board containing gold contact pads, and the heater and the thermopile pads were wire-bonded by aluminum wire for the experiments, as shown in the Figure 8. The temperature distribution on the membrane of the sensor was evaluated using an infrared (IR) camera of InfraTec GmbH, Dresden, Germany with a thermal resolution of <100 bmK, controlled through IRBIS online, version 2.4 software; the data were analyzed by IRBIS plus, version 2.2. To minimize the measurement error caused by the reflection from the surface of the sensor, the measurement was done in a dark room by keeping the camera and the chip in a black box.

To evaluate the TCR (temperature coefficient of resistance) of the heater material, the sensor samples were kept in an oven at different constant temperatures, from 27 °C to 207 °C, at an interval of 10 °C, for a sufficiently long time (2 h), and the resistance of the heater was measured for each constant temperature. The thermal characteristics and the sensitivity of the thermopile were evaluated from the resistance change of the heater (with the TCR curve), as well as with the IR camera. Lastly, the sensitivity of the sensor to hydrogen gas was tested by supplying 2% hydrogen in synthetic air at 200 sccm using a PTFE housing of 1.3 mL volume with an inlet and outlet.

## 3. Results and Discussion

### 3.1. Design Criteria

Table 1 presents a summary of the different design criteria of the sensors used in this work.

**Figure 9:**
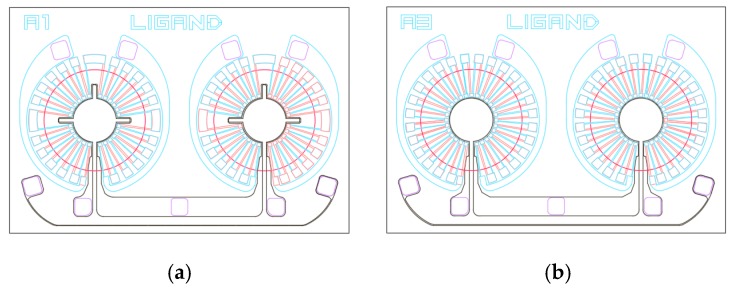
(**a**) Round heater with a loop at the corner (main design A1); (**b**) Round heater without loops (design A3).

In this work, sensor samples of different designs were fabricated. The key aim was to perform a comparative analysis based on the heat loss, power consumption, the uniformity of the temperature field on the membrane, and the thermal sensitivity of the sensor. Among the important performance criteria, several researchers have focused upon a uniform temperature distribution on the membrane, and have applied many ideas, such as placing silicon or polysilicon under the membrane, depositing a metal layer on the membrane, and optimizing the heater design [2]. The first two solutions not only increase the fabrication difficulties, but also the power consumption of the sensor. Therefore, we have optimized the heater design by comparing the performance of two samples, as shown in the Figure 9a,b. Design A1 (main design in this work) has a round heater with three meandering loops at the left, right, and top, while design A3 has a completely round heater without any loop.

Power consumption, another important performance criterion, can be reduced by decreasing the width of the thermoelectric element, which reduces the thermal conduction heat loss from the chip [5,16]. Therefore, the design A2 was fabricated with the half width of the thermocouples (4 µm of WTi and 6 µm of polysilicon), compared to that of the main design A1. A sample (A4) containing a higher number of thermocouples (27 on each thermopile) than the main design (A1) was fabricated to investigate the extent to which the higher number of thermocouples were advantageous. A big issue of debate is whether conduction or convection heat loss is the main contributor to the power consumption of the microsensors. Some literature reveals that convection heat loss dominates over the conduction at high temperature operations, and the convection heat loss coefficient depends on the shape of the micro hotplate’s surface [4,42]. The convection heat loss coefficient for the chip has been discussed in detail in our previous paper [25]. The larger the membrane, the lower the conduction heat loss through the substrate; conversely, a larger membrane causes higher convection heat loss. Therefore, a sample (A5) with a larger membrane (diameter 1000 µm) and a sample (A6) with a smaller membrane (diameter 800 µm) were fabricated to investigate the effect and to optimize the size of the membrane. The most important design criterion is the output of the thermopile that depends on the number of thermocouples connected in series. Conversely, an increased number of thermocouples also increases the conduction power loss. 

### 3.2. Temperature Distribution on the Membrane

**Figure 10:**
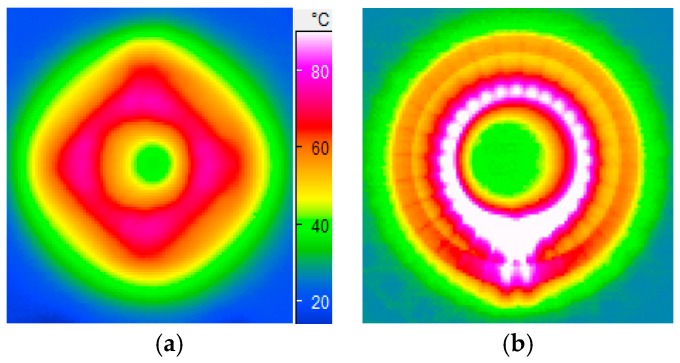
Thermogram for 60 °C average temperature (10V DC supply) (**a**) design A1, maximum and minimum temperatures are 75 °C and 37 °C respectively; (**b**) design A3, maximum and minimum temperatures are 128 °C and 37 °C respectively; the temperature difference is less for A1 than A3.

Figure 10a,b show the thermogram of the membrane of designs A1 and A3, respectively, for a constant 10V DC supply (average temperature ~60 °C). The most important observation is that the temperature variation on the membrane of A3 is much higher than on that of A1. The maximum temperature on A3 reaches 128 °C, and these overheated places may contribute to the deactivation of the catalytic layer. Conversely, the maximum temperature for the design A1 is only 75 °C. The effect of the convection heat loss is higher in the middle of the membrane than on the outer side, as shown in our previous paper [25]. It is evident from the Figure 10a,b that the effect is higher for A3 than A1, creating a larger low temperature region in the middle. High temperature homogeneity is very important for the application of a gas sensor, and the difference between maximum and minimum temperature should not be more than 50 °C [2,43]. For the similar average temperature, the temperature difference on the membrane of A1 is around 3 times lower than that of A3; hence, design A1 provides more homogeneous temperature field. The temperature difference for the design A1 is also much lower (38 °C) than that of the micro hotplate presented by Samaeifar et al. (153 °C) [2].

**Figure 11:**
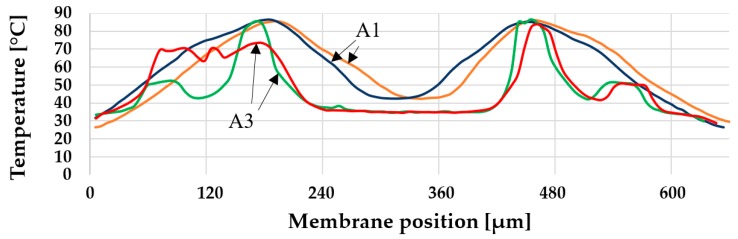
A comparison of temperature profile between design A1 (blue x-axis, yellow y-axis) and A3 (green x-axis, red y-axis); the temperature profile of design A1 is more homogeneous than A3 both along x-axis and y-axis.

A comparison of the temperature profiles of designs A1 and A3 for a specific maximum temperature, 85 °C, (for both x and y-axis) is shown in the Figure 11. There is a profound peak at the corners in both the x- and y-axes profile of design A3, whereas the peak is smoother for design A1. The root mean square deviation (RMSD) from the standard homogeneous temperature profile for designs A1 and A3 are 18.3 °C (20%) and 44.5 °C (50%) respectively.

### 3.3. Thermal Characteristic of the Sensor and the Response in Hydrogen Gas

**Figure 12:**
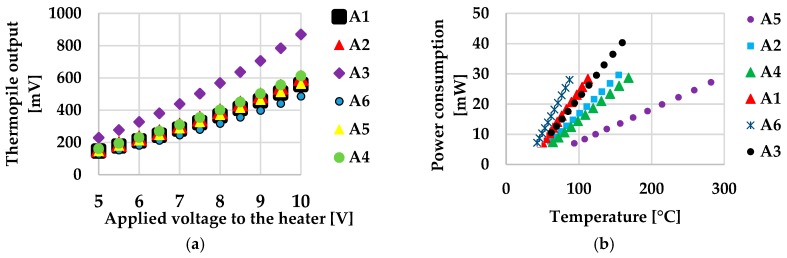
(**a**) Thermopile output of the sensor with applied voltage; (**b**) power consumption of the sensor with heater temperature.

The heater temperature increases with the applied voltage. Figure 12a shows that at a lower applied voltage (lower heater temperature), the thermopile output voltage is almost the same for all the designs. The advantage of the higher number of thermocouples (design A4) over the main design A1 is revealed only at higher voltages, such as at 10V (for a significantly higher heater temperature). The most interesting fact is that at a higher applied voltage, for example, at 10 V, the thermopile output of design A6 is 75 mV lower than that of design A1, although the number of thermocouples is the same. As the membrane size of A6 is smaller, the conduction heat loss through the substrate is higher; consequently, the temperature rise on the membrane is lower than that of A1 for the same applied voltage.

Figure 12b shows the power consumption versus heater temperature for different designs. This study confirms that for almost the same power consumption (around 30 mW), the heater temperature of different designs differs, and thereby provides an idea of the respective thermal loss. For instance, the heater temperature of A6 (smaller membrane) is around 30% lower than that of A1 at the same power consumption, probably due to the higher conduction heat loss through the substrate. It can thus be suggested that the heat loss increases by around 30% (both the conduction and the convection heat loss changes) if the diameter of the membrane decreases by 100 µm (11.11 %). On the other hand, for the same heater temperature (112 °C), the power consumption of A2 is 28.5% lower than that of A1 (main design). The width of the thermopile leads of A2 is half; hence, the conduction heat loss through the thermopile is lower. It is interesting to note that all these effects are higher at higher temperatures than lower temperatures, as there is a profound effect of convection heat loss on the total power consumption which is not linear with the temperature increment. The power consumption of our sensor (A1, main design) is very low, i.e., 14 mW at 90 °C operating temperature.

The generated open circuit voltage in the thermopile due to the Seebeck effect can be expressed by the following formula [11,18,24]:

(1) U=nαΔT

Here, n is the number of thermocouples in the thermopile. The open circuit voltage depends not only on the temperature difference (ΔT) between the junctions of the thermopile, but also on the Seebeck coefficient (α) that is defined by the material characteristics [11]. Although the thermoelectric figure of merit ($\mathrm{ZT}=\alpha^{2}\sigma T/k,$ σ = electrical conductivity, T = temperature, k = thermal conductivity) is strictly defined by the material property, it can be changed by varying the operating temperature [12,13], and the maximum extracted voltage depends on the size and the design of the generator.

Table 2 represents the resistances of the thermopile and the output per thermocouple due to 1 °C temperature change for different designs.

**Figure 13:**
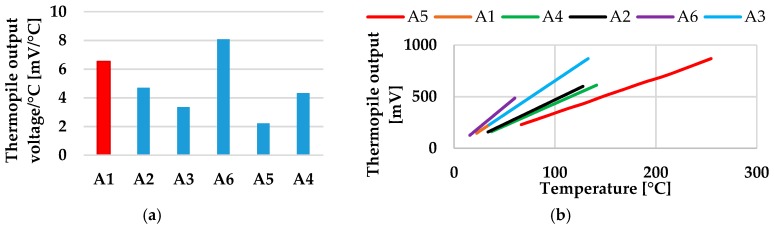
For different sensor designs, (**a**) the thermopile output voltage per 1°C temperature change; (**b**) thermopile output versus temperature difference.

Figure 13a shows that the output of the thermopile due to 1 °C temperature difference between the junctions of the thermopile is 6.54 mV for A1 (main design). Surprisingly, the thermopile output of A4 is the lowest, i.e., 4.33 mV, although it contains a higher number of thermocouples than A1. That may be caused by the higher ohmic drop in the thermopile of A4 due to the higher resistance, which is 18 kΩ higher than A1 (Table 2). Similarly, the output of A2 is also very low, 4.69 mV because the resistance of the thermopile is more than double (126% higher) that of A1, which increases the ohmic drop. Conversely, A6 has very high thermopile output, i.e., 8 mV. A possible cause for this may be that the distance between the cold end and the edge of the membrane is higher than it is in A1. Although the Seebeck coefficient is the same, the output per thermocouple per °C temperature difference varies for different designs. The sensor output (thermopile voltage) increases linearly with the temperature difference between the junctions of the thermopile, regardless of the design; see Figure 13b.

**Figure 14:**
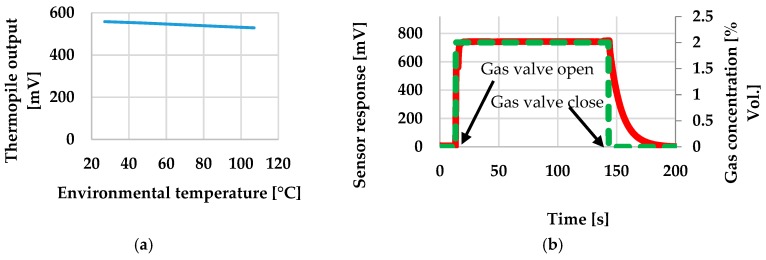
(**a**) Effect of the environmental temperature on the thermopile output; (**b**) the response of the sensor to 2% hydrogen gas.

Figure 14a shows that the sensor output deviates by not more than 5% when the temperature of the environment changes by 100 °C. This is advantageous for the application of a combustible gas sensor, as the environmental temperature change induced by the catalytic combustion will not affect the output of the sensor.

To investigate the performance of the sensor during hydrogen gas detection, a catalytic layer of ligand (p-Phenylenediamine (PDA))-linked platinum nanoparticles network was prepared on one of the membranes. The A1 sensor showed a very high level of response to hydrogen gas compared to the design used in previous similar work [44]. Figure 14b shows the output of the sensor, i.e., 752mV, in 2% hydrogen gas in synthetic air. The response and the recovery time of the sensor are strongly influenced by the sensor housing and the flow rate of the gas [21].

**Figure 15:**
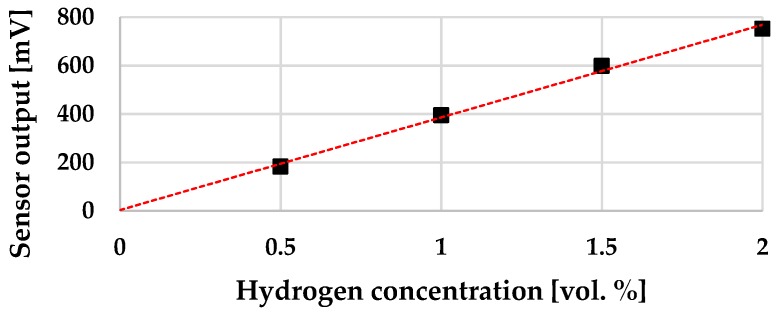
Linear output of the sensor with hydrogen gas concentration.

The sensor with ligand (PDA)-linked Pt nanoparticles catalyst was also tested with different hydrogen gas concentrations. The flowrate and the operating temperature during the experiment were 200 sccm and 130 °C respectively. The output of the sensor was liner with hydrogen gas concentrations (Figure 15).

The sensor with the Pt-PDA catalyst also showed stable output for at least 24 h in a 1.5% continuous hydrogen gas flow at three different operating temperatures (90 °C, 110 °C, and 130 °C) [25]. The safe operating temperature limit of the sensor with a ligand-linked nanoparticles catalyst, such as Pt-PDA, was selected after a couple of experiments, from 90 °C to 130 °C. This is because the catalyst can be deactivated at very high temperatures, due the sintering of the nanoparticles, and at very low temperatures, due to the accumulation of water on the catalyst [25].

The above study confirms that A1 is the optimized design with respect to the homogeneity of the temperature field, power consumption, and the thermopile output voltage; hence, that design was adopted in this work. It was also confirmed that decreasing the width of the thermocouple’s leads not only reduces the power consumption, but also the thermopile output voltage. Although decreasing the size of the membrane increases the thermopile output, it also increases the power consumption of the sensor. One unanticipated finding is that increasing the number of thermocouples, in contrast to our expectations, decreases the thermopile output, while it additionally increases power consumption.

A significant amount of power is consumed by the electronics, i.e., the signal amplifier, booster, or filter, during the operation of the micro sensor [3]. The most interesting feature of our sensor is that the extremely high sensitivity and noise-free signal of the thermopile eliminate the need for these complicated electronic circuits, thereby reducing the power consumption. The thermoelectric output of the sensor presented by Takashiri et al. was only 83.3 mV for 30 °C temperature difference [19], whereas it was almost 200 mV for our sensor. The performance of the sensor in hydrogen was also exceptionally good compared to alternative designs presented by others: e.g., the output of the thermoelectric hydrogen senor presented by Qui et al. was only 3.5 mV for 3% H_2_ [36], and by Nagi et al., was around 0.25 mV for 1.8% H_2_ [21]. Park et al., Huang et al., Shin et al., and Houlet et al. also observed very poor outputs in their thermoelectric sensors, i.e., 3.5 mV, 9 mV, 10 mV, and 60 mV respectively for 1% H_2_ concentration [32,45,46,47]. Our sensor showed 395 mV output for 1% H_2_ concentration, which is about twice the signal that was obtained in similar previous work with ligand-linked, Pt nanoparticles network catalysts, albeit with an old sensor chip design [44].

## 4. Conclusions

We produced a highly sensitive thermoelectric gas sensor with an extremely stable membrane composed of stoichiometric and low-stress SiN. It may be concluded that the developed processes for TiN dry etching with thicker photoresist and WTi wet etching with an AZ 400K developer were crucial for the fabrication of highly sensitive sensors. We found that by adding only 3 thermocouples beyond the optimized design decreases the output of the sensor by around 33%. On the other hand, the power consumption increased by 30% when the membrane diameter was decreased by 11.11 %. The selected optimized design (selected by a comparative performance analysis) shows 6.54 mV/K sensitivity and a response of 752 mV in 2% hydrogen gas in synthetic air.

## Figures and Tables

**Table 1:** Different design criteria.

Design Sample	Design Criteria
A1	Main design (explained in Section 2.1)
A2	Half width of the thermopile structures
A3	Different heater design (round)
A4	Higher number of thermocouples
A5	Larger membrane
A6	Smaller membrane

**Table 2:** Thermopile resistance and the output per thermocouple per °C.

Sensor Type	A1	A2	A3	A4	A5	A6	A1
R Thermopile at 27 °C (kΩ)	194	438	210	212	192	196	194
Output of one thermocouple/°C (µV/K)	273	196	138	160	92.2	336	273
